# Gestational age-specific nomograms for fetal eyeball depth and intraocular measurements using 3-D ultrasound

**DOI:** 10.1007/s00404-026-08541-w

**Published:** 2026-07-22

**Authors:** Marwan Odeh, Rasha Nasra, Zvi Leibovitz, Hasan Bakry, Lior Lowenstein, Inshirah Sgayer

**Affiliations:** 1 Raya Strauss Wing of Obstetrics and Gynecology, Galilee Medical Center, 2210001 Nahariya, Israel; 2 Azrieli Faculty of Medicine, Bar Ilan University, Safed, Israel; 3 Obstetrics and Gynecology Ultrasound Unit, Bnai-Zion Medical Center, Haifa, Israel; 4 Rappaport Faculty of Medicine, Technion-Israel Institute of Technology, Haifa, Israel

**Keywords:** Fetal eye, Ocular biometry, Eyeball depth, Nomograms, Prenatal ultrasound

## Abstract

**Purpose:**

To establish gestational age-specific nomograms for fetal eyeball anteroposterior (AP) diameter and intraocular measurements (lens AP diameter, vitreous body AP diameter).

**Methods:**

A retrospective study was conducted and included singleton pregnancies without prenatal or postnatal structural abnormalities. Three-dimensional ultrasound volumes of the fetal face acquired between 12 and 41 weeks’ gestation were analyzed using multiplanar reformatting to obtain a standardized axial orbital plane. Right and left eyeball, lens, and vitreous body AP diameters were measured. Gestational age-specific reference ranges were generated using percentile distributions. Quadratic regression was used to assess associations with gestational age and biparietal diameter.

**Results:**

Of 608 volumes screened, 588 were included. Complete assessment of all parameters was available in 577 (98.1%). All ocular and intraocular measurements increased with gestational age (all *p* < 0.001). Quadratic regression demonstrated associations between gestational age and eyeball AP diameter (right R^2^ = 0.80; left R^2^ = 0.79), lens AP diameter (right R^2^ = 0.44; left R^2^ = 0.42), and vitreous body AP diameter (right R^2^ = 0.80; left R^2^ = 0.81). Similar associations were observed with biparietal diameter (all *p* < 0.001). Gestational age-specific nomograms and percentile tables are presented.

**Conclusions:**

Gestational age-specific nomograms for fetal eyeball depth and intraocular measurements are provided and may support objective prenatal assessment of fetal ocular growth.

**Supplementary Information:**

The online version contains supplementary material available at https://doi.org/10.1007/s00404-026-08541-w.

## What does this study adds to the clinical work

**Table:** 

This study provides the first gestational age-specific reference ranges for fetal eyeball depth and intraocular dimensions using 3D ultrasound. These noomograms may improve the prenatal evalution of fetal ocular abnormalities, particularly when conventional tranverse orbital measurements appear normal.	

## Introduction

Prenatal ultrasound assessment of the fetal eye has been widely studied, because ocular development reflects both visual system integrity and broader central nervous system maturation [1]. In routine prenatal practice, evaluation of the fetal eye is mainly based on transverse orbital measurements, such as orbital width and interorbital distance, which are useful for confirming the presence of the orbits, assessing symmetry, and identifying craniofacial abnormalities [2, 3]. Additional measurements, including fetal lens diameter and eyeball volume, have been proposed to describe ocular growth [4, 5].

However, these commonly used prenatal measurements do not directly assess the anteroposterior size of the eyeball or allow evaluation of its internal components, such as the lens and vitreous body. As a result, transverse orbital dimensions may remain within normal limits despite abnormal eyeball growth [1, 6, 7]. For example, conditions associated with a small eyeball, such as microphthalmia, or with eyeball enlargement, such as congenital glaucoma, may primarily affect eyeball depth without significantly altering orbital width or interorbital distance, particularly in early or mild cases [8]. Abnormal fetal eyeball size may also have implications beyond visual function, as inadequate eyeball development can impair orbito-facial growth and craniofacial development [9].

In postnatal ophthalmologic practice, ultrasound biometry focuses on measuring eyeball depth and the depth of its internal components, including the lens and vitreous body, which are interpreted using age-specific normative values to assess ocular growth [10–12]. In contrast, prenatal measurements of eyeball depth lack gestational age-specific reference ranges and therefore cannot be objectively interpreted [6, 13]. Thus, a fundamental mismatch exists between prenatal assessment based primarily on transverse orbital measurements and postnatal ophthalmologic evaluation, which relies on axial eyeball length to diagnose and grade abnormalities in eyeball growth.

The objective of this study was to establish gestational age-specific nomograms for fetal eyeball depth, lens depth, and vitreous body depth using three-dimensional ultrasound. By providing normal reference ranges across gestation derived from a large cohort of uncomplicated pregnancies, this study aims to enable objective prenatal assessment of fetal eye growth and improve the detection of abnormal ocular development.

## Methods

### Study design and population

This retrospective observational study was conducted at the Obstetrics and Gynecology Ultrasound Unit, Bnai Zion Medical Center, Haifa, Israel. Consecutive three-dimensional ultrasound volumes of the fetal face acquired between 2009 and 2012 were retrospectively identified and reviewed. This retrospective observational study included singleton pregnancies with three-dimensional ultrasound volumes of the fetal face acquired during the study period. Only examinations from pregnancies with no prenatal or postnatal evidence of fetal structural abnormalities were included, and each fetus contributed a single examination. Ultrasound examinations were performed between 12 and 41 weeks of gestation. Pregnancies were excluded in cases of multiple gestation, fetal growth restriction, prenatally diagnosed fetal structural anomalies, known genetic abnormalities, insufficient image quality precluding reliable ocular measurements, or incomplete clinical or postnatal data.

Pregnancy dating was based on last menstrual period and confirmed by first-trimester crown–rump length measurements. If the two methods were discordant, gestational age was determined according to crown–rump length in accordance with the standard obstetric guidelines [14].

### Ultrasound equipment and image acquisition

All ultrasound examinations were performed using GE Healthcare Voluson 730 Expert and Voluson 730 E8 systems (GE Healthcare, Zipf, Austria). Three-dimensional volume datasets of the fetal face were acquired during routine obstetric ultrasound examinations. Volumes were obtained during periods of fetal quiescence to reduce motion artifacts. All examinations were performed by experts in fetal ultrasound, each with more than 10 years of experience.

### Image reconstruction and plane orientation

**Fig. 1:**
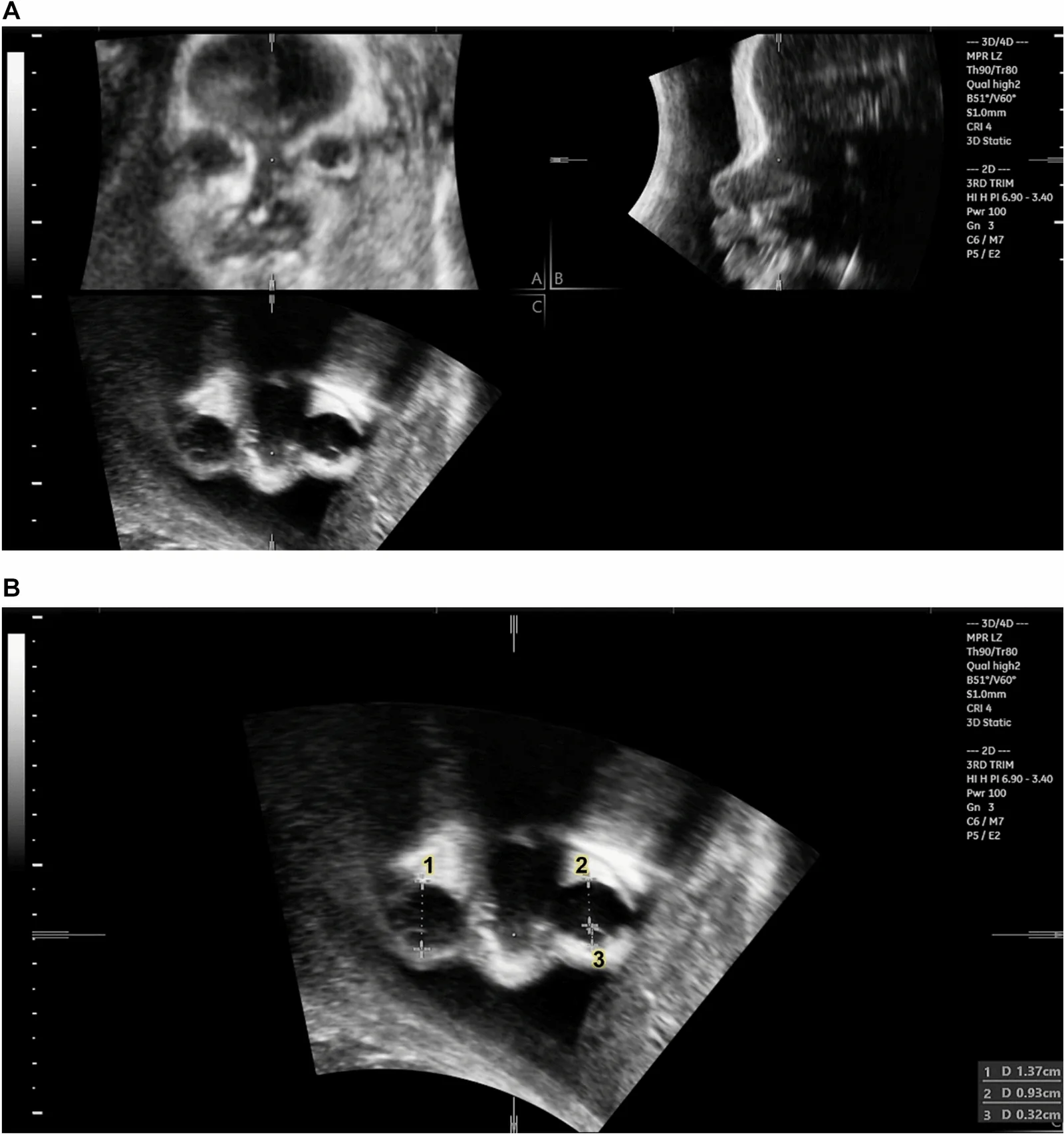
Three-dimensional reconstruction and axial-plane definition for fetal ocular measurements. **a** Multiplanar display of a three-dimensional ultrasound volume of the fetal head, showing simultaneous visualization of the three orthogonal planes. This view enables accurate rotation and alignment of the volume to obtain the true axial (transverse) orbital plane. **b** Three-dimensional reconstructed axial view of the fetal orbits following volume alignment, demonstrating symmetric visualization of both eyeballs and lenses. This reconstructed axial plane was used for all ocular measurements. Shown are the AP diameter of the eyeball (measured from the inner anterior margin of the eyeball to the deepest posterior inner margin), the AP diameter of the lens, and the AP diameter of the vitreous body (measured from the posterior surface of the lens to the posterior inner wall of the eyeball)

Three-dimensional ultrasound volumes of the fetal face were acquired using transabdominal ultrasound, with transvaginal ultrasound performed when required to optimize image quality, and analyzed offline using dedicated multiplanar reconstruction software (4D View, GE Healthcare). Using multiplanar reconstruction, three-dimensional volumes were rotated and aligned to obtain a true axial (transverse) orbital plane (Fig. 1a), confirmed by symmetric visualization of both eyeballs at their maximal transverse diameter; facial bony contours (nasal bridge and zygomatic/maxillary margins) were used as ancillary landmarks when visible (Fig. 1b). All measurements were performed on the reconstructed axial plane. Of 608 volumes initially screened, 588 provided adequate visualization of both eyeballs and sufficient image quality and were included for biometric analysis. Twenty examinations were excluded, because reliable ocular measurements could not be obtained due to inadequate visualization of one or both eyes.

### Ocular measurements

All ocular measurements were obtained on the axial (transverse) plane of the fetal head, with both eyeballs visualized symmetrically at their maximal diameter.

The anteroposterior (A–P) diameter of the eyeball was measured for the right and left eyes from the inner anterior margin of the eyeball to the deepest posterior inner margin.

The lens A–P diameter was measured by placing calipers on the inner anterior and inner posterior lens capsules at the level of maximal lens thickness, ensuring that the measurement passed through the lens center.

The vitreous body A–P diameter was measured from the posterior surface of the lens to the posterior inner wall of the eyeball for the right and left eyes.

Each measurement was obtained twice, and the mean of the two measurements was used for the final analysis.

Standard fetal biometric parameters documented at the time of examination included biparietal diameter (BPD), occipitofrontal diameter (OFD), and head circumference (HC), as measured during routine obstetric assessment.

### Statistical analysis

Measurements were grouped according to gestational-age intervals. For each group, descriptive statistics were calculated, including the number of observations, median values, standard deviation, and percentile distributions (5th, 25th, 75th, and 95th percentiles). Available measurements were included in the corresponding analyses and nomogram construction, whereas cases with missing values for a specific parameter were excluded only from analyses involving that parameter. Nomograms were constructed to describe gestational age-specific reference ranges for each ocular parameter. These nomograms were considered exploratory reference data, based on empirical percentile distributions within gestational-age intervals, rather than definitive population reference standards.

Regression analyses were performed to assess the associations between gestational-age and fetal ocular measurements, including the anteroposterior (AP) diameter of the right and left eyeballs, lens AP diameter, right and left vitreous body AP diameter, as well as their relationships with BPD. Coefficients of determination (r^2^) were calculated for each association. Residual plots were visually inspected to assess model fit and to evaluate major deviations from normality and homoscedasticity after gestational-age correction. A *p* value < 0.05 was considered significant. Statistical analyses were performed using SPSS software version 27.0 (IBM Corp., Armonk, NY).

Internal validation of the quadratic regression models was performed using tenfold cross-validation to assess model stability and reduce the risk of overfitting. Cross-validated R^2^ values were calculated for all models. Statistical analyses and validation procedures were performed using R software (version 4.1.0).

Measurement reproducibility was assessed in a representative random subsample of 30 fetuses. Intra-observer and inter-observer agreement for eyeball AP diameter, lens AP diameter, and vitreous body AP diameter were evaluated using intraclass correlation coefficients (ICCs) with 95% confidence intervals.

## Results

**Fig. 2:**
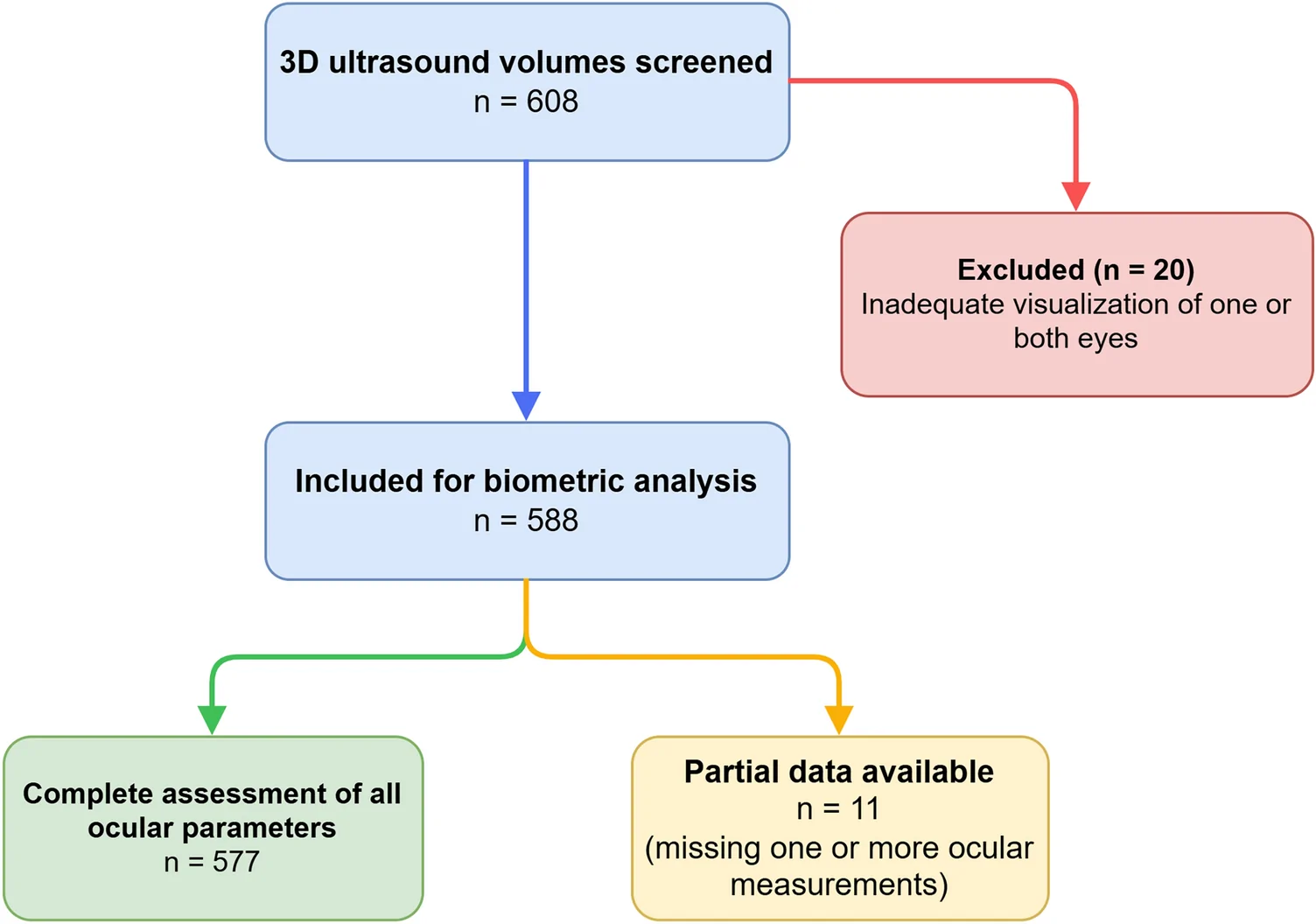
Study flowchart. Selection of ultrasound examinations for inclusion in the study

Of the 608 examinations screened, 20 (3.3%) were excluded, because adequate visualization of one or both eyes could not be obtained for reliable ocular measurements. These image-quality exclusions were distributed relatively evenly across the gestational-age range studied. This left 588 fetuses for analysis; the study selection process is presented in the flowchart (Fig. 2). Of the 588 fetuses included in the analysis, complete assessment of all ocular parameters was achieved in 577 cases (98.1%). In the remaining examinations, partial data were available, including unilateral ocular measurements, isolated vitreous body measurements, or measurements without lens AP diameter assessment.

Gestational age-specific reference ranges for the AP diameter of the right and left eyeballs, lens AP diameter, right and left vitreous body AP diameter are presented in Tables 1,2, 3, 4, 5, 6.

**Table 1:** Nomograms of the right fetal eyeball anteroposterior diameter (mm) according to gestational age

Gestational age	Valid N	Mean	Median	Standard deviation	Percentile 05	Percentile 25	Percentile 75	Percentile 95
12w 1d–15w 0d	13	0.45	0.46	0.06	0.31	0.41	0.5	0.52
15w 1d–16w 0d	37	0.56	0.55	0.08	0.43	0.52	0.62	0.73
16w 1d–17w 0d	43	0.63	0.61	0.08	0.53	0.57	0.7	0.74
17w 1d–22w 0d	19	0.75	0.78	0.15	0.48	0.64	0.88	1.00
22w 1d–23w 0d	22	0.96	0.93	0.23	0.76	0.87	1.01	1.08
23w 1d–24w 0d	110	0.99	0.97	0.14	0.78	0.89	1.07	1.21
24w 1d–25w 0d	111	1.06	1.05	0.14	0.85	0.97	1.12	1.32
25w 1d–26w 0d	24	1.08	1.08	0.13	0.95	1.00	1.12	1.19
26w 1d–29w 0d	25	1.12	1.12	0.1	0.96	1.05	1.18	1.3
29w 1d–32w 0d	30	1.25	1.26	0.1	1.11	1.18	1.32	1.43
32w 1d–33w 0d	31	1.3	1.31	0.15	1.02	1.22	1.36	1.53
33w 1d–34w 0d	23	1.29	1.29	0.08	1.18	1.25	1.35	1.4
34w 1d–37w 0d	39	1.36	1.33	0.1	1.23	1.3	1.43	1.55
37w 1d–40w 0d	22	1.46	1.44	0.16	1.27	1.35	1.58	1.69
40w 1d–42w 0d	5	1.44	1.43	0.04	1.41	1.42	1.43	1.5

**Table 2:** Nomograms of the left fetal eyeball anteroposterior diameter (mm) according to gestational age

Gestational age	Valid N	Mean	Median	Standard deviation	Percentile 05	Percentile 25	Percentile 75	Percentile 95
12w 1d–15w 0d	13	0.46	0.47	0.06	0.36	0.45	0.51	0.53
15w 1d–16w 0d	38	0.56	0.57	0.07	0.45	0.52	0.62	0.66
16w 1d–17w 0d	42	0.63	0.62	0.07	0.52	0.58	0.66	0.73
17w 1d–22w 0d	19	0.77	0.8	0.15	0.5	0.63	0.85	1.05
22w 1d–23w 0d	21	0.93	0.94	0.12	0.76	0.88	0.98	1.03
23w 1d–24w 0d	110	0.99	0.98	0.14	0.8	0.87	1.08	1.26
24w 1d–25w 0d	112	1.05	1.04	0.12	0.88	0.98	1.1	1.25
25w 1d–26w 0d	23	1.08	1.06	0.13	0.92	1	1.1	1.24
26w 1d–29w 0d	26	1.14	1.14	0.11	0.94	1.08	1.18	1.3
29w 1d–32w 0d	29	1.25	1.26	0.1	1.12	1.21	1.33	1.39
32w 1d–33w 0d	31	1.24	1.24	0.24	0.99	1.19	1.39	1.51
33w 1d–34w 0d	24	1.29	1.26	0.13	1.13	1.21	1.35	1.58
34w 1d–37w 0d	39	1.36	1.34	0.1	1.2	1.31	1.4	1.56
37w 1d–40w 0d	18	1.43	1.38	0.21	1.25	1.32	1.44	2.1
40w 1d–42w 0d	5	1.44	1.44	0.14	1.27	1.36	1.46	1.65

**Table 3:** Nomograms of right fetal lens anteroposterior diameter (mm) by gestational age

Gestational age	Valid N	Mean	Median	Standard deviation	Percentile 05	Percentile 25	Percentile 75	Percentile 95
12w 1d–15w 0d	13	0.18	0.17	0.04	0.12	0.16	0.21	0.26
15w 1d–16w 0d	35	0.23	0.23	0.05	0.17	0.2	0.26	0.33
16w 1d–17w 0d	42	0.24	0.23	0.05	0.18	0.21	0.27	0.34
17w 1d–22w 0d	18	0.27	0.25	0.08	0.12	0.22	0.33	0.45
22w 1d–23w 0d	21	0.31	0.31	0.04	0.26	0.29	0.34	0.39
23w 1d–24w 0d	108	0.35	0.33	0.07	0.25	0.3	0.37	0.5
24w 1d–25w 0d	107	0.38	0.37	0.08	0.26	0.33	0.44	0.51
25w 1d–26w 0d	21	0.36	0.36	0.06	0.28	0.31	0.4	0.45
26w 1d–29w 0d	25	0.35	0.34	0.06	0.27	0.31	0.37	0.45
29w 1d–32w 0d	29	0.39	0.38	0.07	0.3	0.36	0.41	0.55
32w 1d–33w 0d	31	0.41	0.41	0.08	0.29	0.37	0.45	0.53
33w 1d–34w 0d	22	0.41	0.39	0.07	0.33	0.35	0.44	0.55
34w 1d–37w 0d	35	0.42	0.41	0.07	0.34	0.37	0.47	0.6
37w 1d–40w 0d	21	0.46	0.41	0.11	0.35	0.39	0.51	0.68
40w 1d–42w 0d	5	0.47	0.48	0.06	0.4	0.42	0.51	0.53

**Table 4:** Nomograms of the left fetal lens anteroposterior diameter (mm) according to gestational age

Gestational age	Valid N	Mean	Median	Standard deviation	Percentile 05	Percentile 25	Percentile 75	Percentile 95
12w 1d–15w 0d	13	0.2	0.2	0.05	0.12	0.17	0.21	0.31
15w 1d–16w 0d	37	0.23	0.23	0.04	0.17	0.2	0.26	0.33
16w 1d–17w 0d	40	0.24	0.24	0.05	0.18	0.2	0.28	0.35
17w 1d–22w 0d	18	0.29	0.27	0.09	0.17	0.22	0.34	0.49
22w 1d–23w 0d	20	0.32	0.32	0.05	0.25	0.29	0.35	0.41
23w 1d–24w 0d	105	0.34	0.33	0.07	0.25	0.3	0.37	0.47
24w 1d–25w 0d	110	0.38	0.37	0.07	0.28	0.33	0.42	0.53
25w 1d–26w 0d	18	0.35	0.35	0.04	0.29	0.33	0.38	0.45
26w 1d–29w 0d	24	0.36	0.35	0.06	0.29	0.31	0.38	0.49
29w 1d–32w 0d	30	0.4	0.4	0.07	0.31	0.36	0.44	0.52
32w 1d–33w 0d	30	0.4	0.39	0.08	0.31	0.34	0.42	0.55
33w 1d–34w 0d	23	0.41	0.38	0.09	0.3	0.33	0.48	0.57
34w 1d–37w 0d	37	0.4	0.4	0.07	0.26	0.37	0.43	0.54
37w 1d–40w 0d	20	0.41	0.41	0.08	0.28	0.37	0.46	0.58
40w 1d–42w 0d	4	0.4	0.41	0.08	0.29	0.35	0.45	0.48

**Table 5:** Nomograms of the right fetal vitreous body anteroposterior diameter (mm) by gestational age

Gestational age	Valid N	Mean	Median	Standard Deviation	Percentile 05	Percentile 25	Percentile 75	Percentile 95
12w 1d–15w 0d	13	0.27	0.27	0.05	0.18	0.26	0.31	0.35
15w 1d–16w 0d	35	0.35	0.36	0.06	0.26	0.29	0.4	0.43
16w 1d–17w 0d	41	0.4	0.38	0.06	0.31	0.36	0.44	0.5
17w 1d–22w 0d	17	0.51	0.51	0.09	0.32	0.44	0.58	0.66
22w 1d–23w 0d	21	0.63	0.62	0.09	0.51	0.57	0.7	0.74
23w 1d–24w 0d	106	0.67	0.65	0.11	0.51	0.6	0.71	0.89
24w 1d–25w 0d	106	0.71	0.7	0.1	0.56	0.62	0.75	0.89
25w 1d–26w 0d	21	0.72	0.74	0.07	0.57	0.7	0.76	0.81
26w 1d–29w 0d	23	0.75	0.76	0.08	0.63	0.69	0.81	0.89
29w 1d–32w 0d	28	0.87	0.89	0.09	0.73	0.83	0.93	0.99
32w 1d–33w 0d	31	0.88	0.88	0.1	0.62	0.84	0.94	1.03
33w 1d–34w 0d	22	0.9	0.9	0.09	0.75	0.85	0.94	1.06
34w 1d–37w 0d	35	0.93	0.92	0.11	0.74	0.87	1	1.15
37w 1d–40w 0d	21	1.03	1	0.13	0.87	0.96	1.09	1.31
40w 1d–42w 0d	5	1.02	1	0.05	0.97	0.98	1.03	1.1

**Table 6:** Nomograms of the left fetal vitreous body anteroposterior diameter (mm) according to gestational age

Gestational age	Valid N	Mean	Median	Standard deviation	Percentile 05	Percentile 25	Percentile 75	Percentile 95
12w 1d–15w 0d	13	0.27	0.27	0.05	0.19	0.23	0.3	0.38
15w 1d–16w 0d	37	0.35	0.35	0.05	0.25	0.3	0.38	0.44
16w 1d–17w 0d	39	0.39	0.4	0.06	0.3	0.34	0.43	0.53
17w 1d–22w 0d	18	0.51	0.51	0.09	0.36	0.43	0.59	0.66
22w 1d–23w 0d	20	0.62	0.61	0.1	0.47	0.54	0.69	0.8
23w 1d–24w 0d	102	0.67	0.65	0.13	0.51	0.6	0.72	0.87
24w 1d–25w 0d	109	0.68	0.68	0.09	0.54	0.61	0.74	0.8
25w 1d–26w 0d	18	0.72	0.7	0.06	0.62	0.67	0.77	0.85
26w 1d–29w 0d	23	0.77	0.78	0.11	0.62	0.7	0.83	0.93
29w 1d–32w 0d	28	0.87	0.87	0.07	0.77	0.82	0.92	0.99
32w 1d–33w 0d	30	0.88	0.9	0.11	0.66	0.82	0.94	1.04
33w 1d–34w 0d	23	0.88	0.89	0.09	0.72	0.82	0.95	0.99
34w 1d–37w 0d	37	0.95	0.97	0.1	0.77	0.91	1	1.11
37w 1d–40w 0d	18	1.01	1.02	0.1	0.82	0.94	1.08	1.19
40w 1d–42w 0d	4	1.05	1.06	0.13	0.9	0.96	1.15	1.2

Quadratic regression analyses demonstrated a significant positive association between GA and all orbital and intraocular measurements (all with *p* < 0.001). Right and left eyeball anteroposterior diameters (Supplementary Figs. 1a, b) showed a significant increase with GA (right: R^2^ = 0.80, *y* = − 0.657 + 0.098 × GA − 0.00115 × GA^2^; left: R^2^ = 0.79, *y* = − 0.634 + 0.097 × GA − 0.00113 × GA^2^). Lens anteroposterior diameter also demonstrated a significant association with GA (right: R^2^ = 0.44, *y* = − 0.133 + 0.0298 × GA − 0.000392 × GA^2^ (Supplementary Fig. 1c); left: R^2^ = 0.42, *y* = − 0.178 + 0.0348 × GA − 0.000514 × GA^2^ (Supplementary Fig. 1d)). Vitreous body anteroposterior diameter increased with GA following a quadratic pattern on both sides (right: R^2^ = 0.80, *y* = − 0.500 + 0.0680 × GA − 0.000757 × GA^2^ (Supplementary Fig. 1e); left: R^2^ = 0.81, *y* = − 0.482 + 0.0657 × GA − 0.000708 × GA^2^ (Supplementary Fig. 1f)).

Regression analyses demonstrated a significant association between BPD and all orbital and intraocular measurements (all p < 0.001). Right and left eyeball diameters showed a significant increase with BPD (right: R^2^ = 0.79, y = − 0.120 + 0.246 × BPD − 0.00857 × BPD^2^; left: R^2^ = 0.76, y = − 0.128 + 0.252 × BPD − 0.00954 × BPD^2^). Lens anteroposterior diameter also demonstrated a significant association with BPD (right: R^2^ = 0.43, y = 0.023 + 0.0769 × BPD − 0.00347 × BPD^2^; left: R^2^ = 0.39, y = − 0.0046 + 0.0921 × BPD − 0.00515 × BPD^2^). Vitreous body anteroposterior diameter increased with BPD on both sides (right: R^2^ = 0.79, y = − 0.095 + 0.159 × BPD − 0.00447 × BPD^2^; left: R^2^ = 0.79, y = − 0.112 + 0.163 × BPD − 0.00472 × BPD^2^).

Cross-validated R^2^ values were highly similar to the original model R^2^ values for all parameters, supporting the robustness and stability of the proposed nomograms (Supplementary Tables 1 and 2).

Measurement reproducibility was assessed in a representative subsample of 30 fetuses. Both intra-observer and inter-observer analyses demonstrated good-to-excellent agreement for all ocular measurements. Detailed ICC values and 95% confidence intervals are presented in Supplementary Tables 3 and 4.

Application of AP nomograms in fetuses with microphthalmia.

**Fig. 3:**
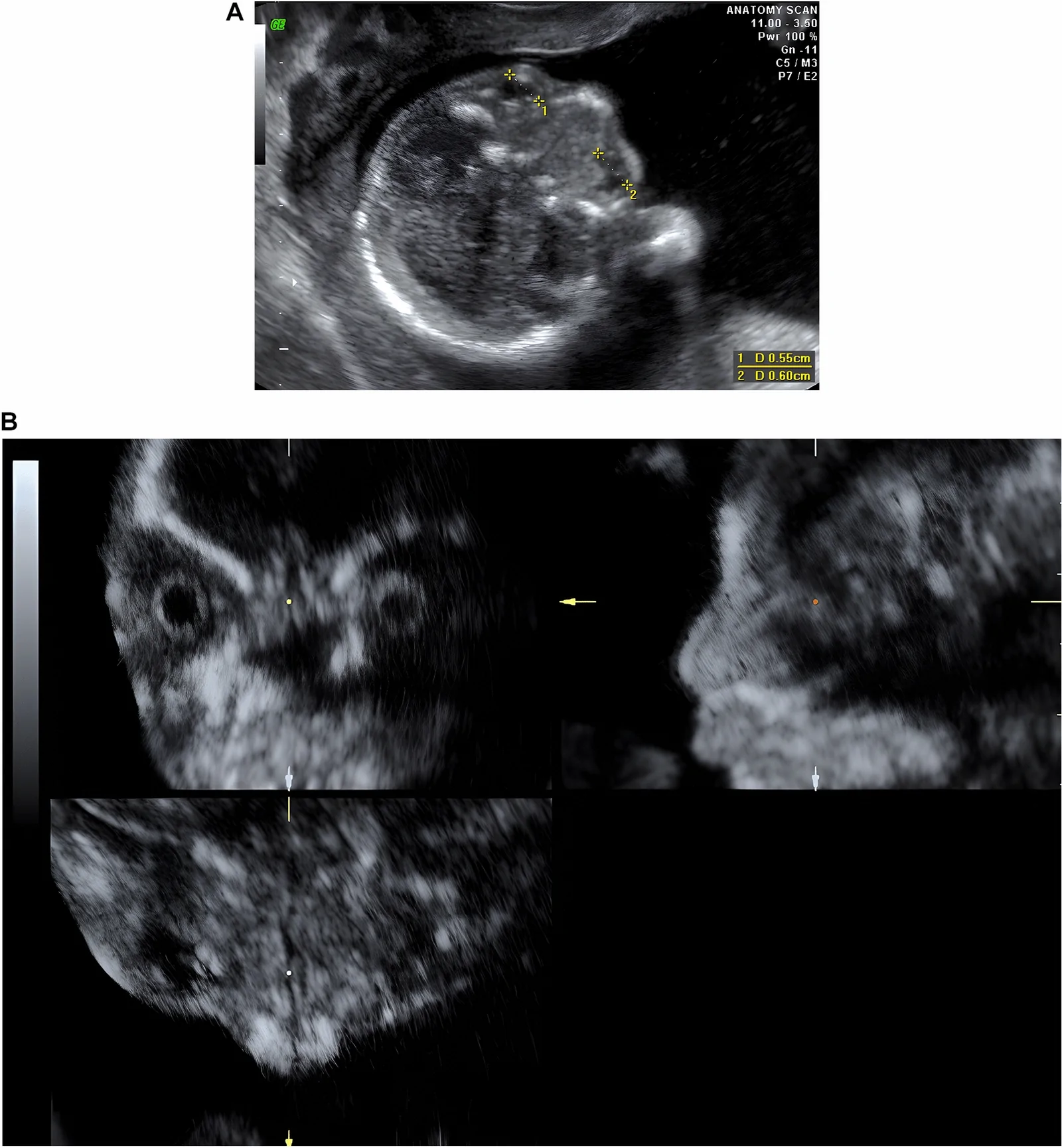
Axial three-dimensional reconstructed ultrasound images of the fetal orbits at 18 weeks’ gestation. **a** The upper panel demonstrates preserved transverse (lateral) orbital diameters. **b** The lower panels illustrate reduced anteroposterior eyeball diameters in the axial orbital plane, consistent with bilateral microphthalmia. Transverse orbital width was within the normal reference range for gestational age according to published charts

Four fetuses with microphthalmia were evaluated using the proposed anteroposterior (AP) nomograms and compared with transverse orbital percentiles according to Goldstein reference charts [15] (Fig. 3). In Fetus 1, examined at 15 weeks’ gestation, right microphthalmia was identified, with a right eyeball AP diameter of 0.36 cm corresponding to a z-score of − 2.86, while transverse orbital width was at the 25th percentile. In Fetus 2, examined at 23.6 weeks’ gestation, right microphthalmia was observed, with a right eyeball AP diameter of 0.64 cm and a corresponding z-score of − 2.5; transverse orbital width was at the 10th percentile. In Fetus 3, evaluated at 15 weeks’ gestation, bilateral microphthalmia was present, with a right eyeball AP diameter of 0.31 cm and corresponding z-scores of − 3.71 for the right eye and − 2.86 for the left eye, while transverse orbital width was at the 25th percentile. In Fetus 4, assessed at 17.5 weeks’ gestation, bilateral microphthalmia was identified, with an eyeball AP diameter of 0.38 cm corresponding to bilateral z-scores of − 2.31, and transverse orbital width was at the 25th percentile for gestational age. Figure 3a demonstrates preserved transverse orbital diameters, whereas Fig. 3b illustrates the reduced anteroposterior eyeball dimensions. All four pregnancies were terminated following prenatal diagnosis. Therefore, postnatal ophthalmologic assessment was not available, and no postmortem examination was performed.

## Discussion

In this study, we extended fetal eye biometry by establishing gestational age-specific sonographic nomograms for the fetal AP eyeball diameter and related intraocular measurements using three-dimensional ultrasound in the axial plane. Measurements were feasible across a wide gestational-age range and demonstrated consistent growth patterns, supporting their use as exploratory normative reference data.

Prenatal evaluation of suspected microphthalmia often relies on transverse orbital measurements and/or a single axial eyeball dimension, implicitly assuming that ocular size abnormalities will be reflected uniformly in these parameters. However, microphthalmia is developmentally heterogeneous and may involve disproportionate reduction of specific intraocular components. Component-level AP assessment may therefore be more sensitive to early or segment-predominant forms, particularly when orbital width remains preserved. As described by Ragge et al., microphthalmia encompasses a spectrum of phenotypes and may preferentially involve the anterior segment, the posterior segment, or a combination of both, each arising from different underlying mechanisms [9]. In such cases, abnormal ocular size may result predominantly from a reduced vitreous body, a small lens, or disproportionate shortening of the posterior segment, while other components may be relatively preserved [9]. Consequently, reliance on a single axial measurement may underestimate or fail to detect abnormal ocular development, particularly when posterior-segment involvement predominates.

This limitation is further supported by longitudinal postnatal data, indicating that orbital width does not reliably reflect intraocular growth. In a prospective ultrasonographic cohort of children with congenital microphthalmia, Groot et al. followed changes in eye AP diameter together with orbital width over time [8]. They demonstrated that these parameters may evolve differently: while eye AP diameter increased in some affected eyes, remained stable in others, or decreased over time, orbital width often remained relatively preserved, with many unilateral cases maintaining orbital symmetry at follow-up [8]. Moreover, eyes with similar orbital widths could exhibit divergent AP growth trajectories and shifts in severity classification.

Our findings complement the prenatal orbital charts reported by Feldman et al., who proposed gestational age-specific reference intervals for fetal total axial length (TAL) measured in the axial plane [16]. As both TAL and the eyeball anteroposterior diameter assessed in the present study reflect the axial dimension of the fetal globe, direct comparison is possible. Feldman et al. reported a strong correlation between TAL and gestational age (R^2^ = 0.89), while our study demonstrated similarly strong correlations for eyeball anteroposterior diameter (R^2^ = 0.79–0.80). Earlier prenatal work by Achiron et al. evaluated intraocular structures using circumferential indices of the lens and vitreous body rather than direct anteroposterior dimensions [6]. In contrast, the present study provides gestational age-specific nomograms for eyeball anteroposterior diameter together with separate anteroposterior measurements of the lens and vitreous body, enabling component-level assessment of ocular development. Taken together, these observations support the value of complementary anteroposterior ocular measurements beyond orbital width alone to achieve a more comprehensive prenatal evaluation.

To illustrate potential clinical utility, we applied the proposed anteroposterior nomograms to four fetuses with confirmed microphthalmia. In all cases, eyeball AP diameter was markedly reduced (approximately ≤ − 2 SD), while transverse orbital width remained within the normal range or only mildly reduced based on commonly used reference charts. This discordance highlights a potential limitation of relying on transverse orbital measures alone and suggests that axial, component-level assessment may provide complementary information when microphthalmia is suspected, particularly in early gestation or milder phenotypes where orbital width may appear preserved. Although these cases are few and were not intended to define diagnostic thresholds, they provide a descriptive illustration of how the proposed nomograms may be applied in fetuses with microphthalmia. Further studies are required to determine their diagnostic performance and clinical utility. The use of a clearly defined measurement approach that can be applied consistently in both prenatal and postnatal ultrasound examinations, obtained in a reproducible imaging plane and interpreted using uniform reference charts, enables evaluation of the same anatomical structures within a shared framework. This continuity between prenatal and postnatal assessment promotes clearer counseling, supports meaningful longitudinal comparison, and minimizes uncertainty arising from the use of disparate parameters at different stages. Among our cohort, the eyeball AP diameter, lens AP diameter, and vitreous body AP diameter were highly feasible to obtain, with successful measurement in 99.0% of examinations between 14 and 35 weeks of gestation. We therefore suggest that these measurements may be particularly valuable in targeted assessments when an ocular abnormality is suspected, rather than as part of routine screening. Although gestational age-based nomograms are expected to be the primary reference in clinical practice, the BPD-based nomograms may provide complementary information in cases of uncertain pregnancy dating or when ocular measurements are interpreted in the context of overall fetal biometry. Although all parameters demonstrated significant associations with gestational age, the relationship was stronger for eyeball and vitreous body measurements than for lens AP diameter, suggesting that the former may provide more robust markers of ocular growth across gestation.

This study is strengthened by the application of three-dimensional ultrasound with multiplanar reformatting, which allowed reliable visualization of the true axial plane of the fetal orbits and consistent bilateral measurements. By examining ocular and intraocular dimensions across gestation in a well-dated cohort with normal neonatal outcomes, we provide reference data that are directly applicable to prenatal imaging. Limitations include the observational design and the acquisition of measurements at a single time point, as well as dependence on image quality and fetal position, particularly in late gestation. Moreover, gestational-age intervals were not uniform and were adjusted according to the number of available examinations within each gestational-age range to ensure adequate sample sizes for percentile estimation. As a result, some intervals were wider than others, which may have reduced temporal resolution in certain portions of gestation. In addition, the distribution of examinations was not uniform across gestation, with fewer observations at the extremes of gestational age and a greater concentration of examinations during mid-gestation, which may have reduced the precision of percentile estimates in underrepresented gestational-age ranges. Accordingly, the nomograms are expected to be most reliable within gestational-age ranges containing the highest density of observations, whereas percentile estimates at the extremes of gestation should be interpreted with greater caution. Therefore, the proposed nomograms should be regarded as exploratory and require external validation before being adopted as definitive fetal biometric reference standards. All available gestational ages were retained to provide a complete descriptive overview of fetal ocular growth across the studied gestational-age range. Moreover, fetal sex was not consistently available in the study database and therefore could not be evaluated as a potential determinant of ocular biometry. A further limitation is that the microphthalmia cases represent a small, descriptive proof-of-concept sample; therefore, the present data cannot establish diagnostic cutoffs or estimate sensitivity/specificity, and larger cohorts with standardized postnatal ophthalmologic correlation are required. In addition, all examinations were performed using older-generation ultrasound systems. Although the measurements were based on reproducible anatomical landmarks identified through multiplanar reconstruction, differences in image quality and volume acquisition capabilities compared with contemporary ultrasound platforms may affect the generalizability of the reference ranges. Validation using current-generation ultrasound systems would therefore be valuable. Moreover, the relative position of each orbit to the ultrasound transducer at the time of volume acquisition was not recorded. Therefore, potential near-field/far-field effects, which may influence image resolution and measurement precision despite multiplanar reconstruction, could not be evaluated and may have contributed to variability between right- and left-sided measurements.

In conclusion, we established gestational age-specific nomograms for fetal eyeball AP diameter and individual intraocular components, including the lens and vitreous body, using three-dimensional ultrasound. These data provide a component-level framework for prenatal assessment of ocular development that extends beyond reliance on axial length alone. The presented reference ranges may provide a framework for the objective characterization of abnormal fetal ocular growth; however, their clinical utility remains hypothesis-generating and requires validation in larger prospective studies. Early prenatal recognition of fetal abnormalities is important for parental counseling, pregnancy management, and perinatal planning [17]. Future studies should focus on validating these nomograms in abnormal eyes, correlating prenatal deviations with postnatal ophthalmologic findings, and exploring the clinical relevance of right–left asymmetry.

## Supplementary Information

Below is the link to the electronic supplementary material.

Supplementary file4 (DOCX 13 KB)

Supplementary file1 (PNG 14 KB)

Supplementary file2 (PNG 15 KB)

Supplementary file3 (PNG 16 KB)

Supplementary file6 (PNG 15 KB)

Supplementary file7 (PNG 13 KB)

Supplementary file8 (PNG 14 KB)

Supplementary file9 (PNG 14 KB)

Supplementary file10 (PNG 15 KB)

Supplementary file11 (PNG 16 KB)

Supplementary file12 (PNG 15 KB)

Supplementary file13 (PNG 13 KB)

Supplementary file14 (PNG 14 KB)

Supplementary file5 (DOCX 13 KB)

## Data Availability

The data that support the findings of this study are not publicly available due to their containing information that could compromise the privacy of the research participants but are available from the corresponding author upon reasonable request.
