# Supplementary material for: Gestational age-specific nomograms for fetal eyeball depth and intraocular measurements using 3-D ultrasound
Source: Arch Gynecol Obstet. 2026 Jul 22;313(1):295. doi: 10.1007/s00404-026-08541-w (PMC13677642; doi:10.1007/s00404-026-08541-w)
Supplement: Supplementary file 1 [file 404_2026_8541_MOESM14_ESM.docx]

**Supplementary Table 1.**

**Original and cross-validated R² values for gestational age (GA)-based quadratic regression models of fetal ocular and intraocular measurements.**

|  | Original R^2^ | Cross-Validated R^2^ |
| --- | --- | --- |
| BOD | 0.9272 | 0.9264 |
| BID | 0.8222 | 0.8208 |
| ORBIT AP RT | 0.8000 | 0.7986 |
| ORBIT AP LT | 0.7910 | 0.7890 |
| LENS AP RT | 0.4420 | 0.4357 |
| LENS AP LT | 0.4201 | 0.4126 |
| INTER LENS DISTANCE | 0.8854 | 0.8846 |
| VITEROUS AP RT | 0.8011 | 0.7988 |
| VITEROUS AP LT | 0.8051 | 0.8041 |

**Independent Variable GA*

**Supplementary Table 2.**

**Original and cross-validated R² values for biparietal diameter (BPD)-based quadratic regression models of fetal ocular and intraocular measurements.**

|  | Original R^2^ | Cross-Validated R^2^ |
| --- | --- | --- |
| BOD | 0.9091 | 0.9127 |
| BID | 0.8088 | 0.8177 |
| ORBIT AP RT | 0.7875 | 0.7930 |
| ORBIT AP LT | 0.7610 | 0.7676 |
| LENS AP RT | 0.4284 | 0.4374 |
| LENS AP LT | 0.3909 | 0.4030 |
| INTER LENS DISTANCE | 0.8577 | 0.8622 |
| VITEROUS AP RT | 0.7918 | 0.7968 |
| VITEROUS AP LT | 0.7888 | 0.7951 |

**Independent Variable BPD*
