# Supplementary material for: Gestational age-specific nomograms for fetal eyeball depth and intraocular measurements using 3-D ultrasound
Source: Arch Gynecol Obstet. 2026 Jul 22;313(1):295. doi: 10.1007/s00404-026-08541-w (PMC13677642; doi:10.1007/s00404-026-08541-w)
Supplement: Supplementary file 2 [file 404_2026_8541_MOESM1_ESM.docx]

| **Parameter** | **ICC (95% CI)** | **p-value** | **Interpretation** |
| --- | --- | --- | --- |
| Parameter 1 | 0.880 (0.763–0.941) | <0.001 | Good |
| Parameter 2 | 0.922 (0.842–0.962) | <0.001 | Excellent |
| Parameter 3 | 0.784 (0.594–0.891) | <0.001 | Good |
| Parameter 4 | 0.807 (0.633–0.903) | <0.001 | Good |
| Parameter 5 | 0.906 (0.812–0.954) | <0.001 | Excellent |
| Parameter 6 | 0.826 (0.666–0.913) | <0.001 | Good |

| Measurement 1 | 0.846 (0.701–0.924) | <0.001 | Good |
| --- | --- | --- | --- |
| Measurement 2 | 0.797 (0.619–0.897) | <0.001 | Good |
| Measurement 3 | 0.777 (0.584–0.887) | <0.001 | Good |
| Measurement 4 | 0.821 (0.660–0.911) | <0.001 | Good |
| Measurement 5 | 0.845 (0.703–0.923) | <0.001 | Good |
| Measurement 6 | 0.760 (0.508–0.885) | <0.001 | Good |
|  |  |  |  |
